# Chronic Adipose Tissue Inflammation Linking Obesity to Insulin Resistance and Type 2 Diabetes

**DOI:** 10.3389/fphys.2019.01607

**Published:** 2020-01-29

**Authors:** Federica Zatterale, Michele Longo, Jamal Naderi, Gregory Alexander Raciti, Antonella Desiderio, Claudia Miele, Francesco Beguinot

**Affiliations:** ^1^Department of Translational Medicine, University of Naples Federico II, Naples, Italy; ^2^URT Genomic of Diabetes, Institute of Experimental Endocrinology and Oncology, National Research Council, Naples, Italy; ^3^Department of Environmental, Biological, and Pharmaceutical Sciences and Technologies, University of Campania Luigi Vanvitelli, Caserta, Italy

**Keywords:** obesity, insulin resistance, diabetes, low-grade inflammation, adipose tissue inflammation, innate immune system, adaptive immunity, inflammatory triggers

## Abstract

Obesity is one of the major health burdens of the 21st century as it contributes to the growing prevalence of its related comorbidities, including insulin resistance and type 2 diabetes. Growing evidence suggests a critical role for overnutrition in the development of low-grade inflammation. Specifically, chronic inflammation in adipose tissue is considered a crucial risk factor for the development of insulin resistance and type 2 diabetes in obese individuals. The triggers for adipose tissue inflammation are still poorly defined. However, obesity-induced adipose tissue expansion provides a plethora of intrinsic signals (e.g., adipocyte death, hypoxia, and mechanical stress) capable of initiating the inflammatory response. Immune dysregulation in adipose tissue of obese subjects results in a chronic low-grade inflammation characterized by increased infiltration and activation of innate and adaptive immune cells. Macrophages are the most abundant innate immune cells infiltrating and accumulating into adipose tissue of obese individuals; they constitute up to 40% of all adipose tissue cells in obesity. In obesity, adipose tissue macrophages are polarized into pro-inflammatory M1 macrophages and secrete many pro-inflammatory cytokines capable of impairing insulin signaling, therefore promoting the progression of insulin resistance. Besides macrophages, many other immune cells (e.g., dendritic cells, mast cells, neutrophils, B cells, and T cells) reside in adipose tissue during obesity, playing a key role in the development of adipose tissue inflammation and insulin resistance. The association of obesity, adipose tissue inflammation, and metabolic diseases makes inflammatory pathways an appealing target for the treatment of obesity-related metabolic complications. In this review, we summarize the molecular mechanisms responsible for the obesity-induced adipose tissue inflammation and progression toward obesity-associated comorbidities and highlight the current therapeutic strategies.

## Introduction

Overweight and obesity are the consequence of a chronic imbalance between energy intake and energy expenditure, culminating in the excess of fat accumulation in AT. Since 1980, the global incidence of overweight and obesity has risen to the extent that almost one-third of the world population is now considered being overweight or obese (Chooi et al., 2019).

Obesity is a heterogeneous condition deriving from genetic and lifestyle interactions (Albuquerque et al., 2015; Hopkins and Blundell, 2016; MacLean et al., 2017; Schwartz et al., 2017) and is correlated with several pathological dysfunctions with important repercussions for individual and community health (Kyrou et al., 2018). Lifestyle and behavioral interventions (e.g., increased physical activity and decreased caloric intake) are fundamental parts for weight control (Butryn et al., 2011; Greenway, 2015). A gradual weight loss up to 16% of the original body weight is sufficient to improve β-cell function and insulin sensitivity in AT, liver, and skeletal muscle (Magkos et al., 2016). The improved glycemic control after weight loss is due, in part, to the dysregulated expression of genes involved in cholesterol flux, lipid synthesis, ECM remodeling, and oxidative stress (Magkos et al., 2016). In the light of the foregoing, obesity is the most frequent metabolic disorder in the world and the primary risk factor for IR and diabetes mellitus (Boles et al., 2017).

Diabetes mellitus refers to a group of conditions in which the body cannot use and store glucose correctly (American Diabetes Association, 2018). The proportion of individuals affected by diabetes mellitus has risen dramatically over the previous three decades, making it one of the major causes of death in the world. More than 300 million people are expected to develop T2D as a complication of obesity by 2025 (Ncd Risk Factor Collaboration [Ncd-RisC], 2016).

T2D is the most prevalent form of diabetes mellitus (American Diabetes Association, 2018), a chronic disease characterized by increased plasma glucose levels due to insulin secretion deficiencies (i.e., β-cell dysfunction) and IR (i.e., decreased target tissue capacity to react regularly to insulin) (American Diabetes Association, 2018).

Obesity is a risk factor for IR and a complete understanding of the mechanisms linking obesity to IR will enhance our knowledge of T2D pathogenesis and the capacity to manage obesity-related disorders (Choi and Cohen, 2017; Maksymets et al., 2018). For this purpose, several studies have been conducted on human and transgenic animal models demonstrating a correlative and causative association between dietary excess and activation of the innate and adaptive immune system in organs that control systemic energy homeostasis (Lumeng et al., 2007b; Lumeng and Saltiel, 2011; Lackey and Olefsky, 2016).

The initial mechanistic evidence supporting the inflammatory origin of obesity and diabetes comes from human and animal studies carried out in the early 1990s. In these studies, AT from obese rodents and humans show inflammatory modifications and enhanced secretion of pro-inflammatory cytokine TNF-α able to induce IR by inactivating the IRS-1 (Hotamisligil et al., 1993, 1995; Uysal et al., 1998). The pivotal role of TNF-α is significantly supported by evidence establishing that TNF-α neutralization in obese mice improves insulin sensitivity and glucose metabolism (Hotamisligil et al., 1993).

Low-grade chronic AT inflammation (also noted as meta-inflammation) is strongly and consistently associated with excess body fat mass and is characterized by infiltration and activation of pro-inflammatory macrophages and other immune cells that produce and secrete pro-inflammatory cytokines and chemokines (Chawla et al., 2011; Burhans et al., 2018).

Macrophages change not only their number during obesity (i.e., up to 40% of all AT cells in this context) but also their location and inflammatory phenotype (Weisberg et al., 2003). While in normal weight subjects the macrophages show anti-inflammatory properties, the polarization of AT macrophages (ATMs) in obese AT shifts to a pro-inflammatory phenotype (Lumeng et al., 2007a; Castoldi et al., 2016; Boulenouar et al., 2017). In obesity, macrophages surround dead adipocytes (i.e., forming crown-like structures) and secrete an array of pro-inflammatory cytokines that lead to local and systemic inflammation and IR (Lumeng et al., 2007a; Haase et al., 2014).

The inflammatory triggers are still almost unknown; however, obesity-induced AT remodeling provides a plethora of intrinsic signals (e.g., adipocyte death, hypoxia, and mechanical stress) capable of initiating an inflammatory response (Reilly and Saltiel, 2017). The role of inflammation in T2D pathogenesis and associated metabolic complications has led to a growing interest in targeting inflammatory mediators or pathways to prevent and treat T2D (Shoelson et al., 2006; McLaughlin et al., 2016).

In this review, we address the primary role played by the loss of immune regulation in the AT inflammation and the development of obesity-associated disorders, providing details on molecular aspects. We highlight the cellular and molecular triggers for obesity-induced inflammation and finally give some insights into the new anti-inflammatory therapeutic strategies.

## Molecular Pathways Linking Obesity-Induced Inflammation and Ir

Insulin is an anabolic hormone secreted by β-cells that plays a crucial role not only in carbohydrate metabolism but also in protein and lipid anabolic regulation, cell growth, and proliferation (Fu et al., 2013). Blood glucose concentrations stimulate insulin synthesis and release; its effects on whole-body metabolism result from its binding to the cell membrane receptor, which is activated by autophosphorylation of specific tyrosine residues. The activated insulin receptor phosphorylates and recruits intracellular proteins, also known as IRSs. Downstream of IRS proteins, PI3K mediates insulin functions mainly by activating PKB and protein kinase C cascades (i.e., stimulating glucose uptake, glycogen synthesis and inhibiting hepatic gluconeogenesis). Insulin signaling also exerts mitogenic effects, most of which are mediated by PKB cascade and Ras/mitogen activated protein kinase (MAPK) pathway activation (Ramalingam et al., 2013).

Obesity association with T2D has long been recognized, and the primary reason is the ability of obesity to promote IR, the main pathophysiological aspect of T2D (Kahn and Flier, 2000).

IR is a metabolic complication in which the three major insulin-sensitive tissues (skeletal muscle, liver, and AT) become less responsive to insulin action. IR is characterized by serious failures in glucose uptake, glycogen synthesis, and, to a lesser extent, glucose oxidation (Ormazabal et al., 2018). In this scenario, the β-cells compensate for IR by increasing insulin secretion and restoring blood glucose concentration within the normal range. A further decline in insulin sensitivity makes the β-cells exhausted, and this results in persistent hyperglycemia and T2D (Shulman, 2000).

A number of studies have been performed to identify the causal factors responsible for obesity-induced IR. One of the most accepted theories considers chronic systemic inflammation induced by obesity as a preponderant mechanism (Weisberg et al., 2003; Xu et al., 2003; Luft et al., 2013). This theory is strongly supported by many findings and clinical evidence; for instance, inflammatory markers such as CRP, TNF-α, and interleukin 6 (IL-6) are elevated in obese and insulin-resistant subjects (Dandona et al., 1998; Kern et al., 2001; Vozarova et al., 2001; Phosat et al., 2017; Uemura et al., 2017). The first evidence of an association between IR and inflammation has been hypothesized when, following the administration of anti-inflammatory agents, an improvement in glucose homeostasis has been observed in T2D patients (Williamson, 1901; Reid et al., 1957; Yuan et al., 2001; Hundal et al., 2002). Further studies in the mid-1990s have shown that the white AT (WAT) of obese rodents and humans exhibited changes in the levels of pro-inflammatory molecules (e.g., TNF-α) (Hotamisligil et al., 1993, 1995; Uysal et al., 1998). Such inflammatory mediators modulate IR either directly by affecting insulin signaling or indirectly by stimulating inflammatory pathways (Tilg and Moschen, 2008). Other studies have shown that hypoxia, which occurs in AT during obesity, is directly responsible for IR induction in both human and murine models (Regazzetti et al., 2009; Yin et al., 2009).

Animal and human studies have identified WAT as the primary site where obesity-related chronic inflammation is initiated and exacerbated (Weisberg et al., 2003; Xu et al., 2003). AT remodeling during obesity provides a plethora of intrinsic and extrinsic signals capable of triggering an inflammatory response (Chawla et al., 2011; Huh et al., 2014; Reilly and Saltiel, 2017). These triggers, discussed later in the review, converge on the activation of the JNK and NF-κB signaling pathways (Nakatani et al., 2004; Shoelson et al., 2006; Bluher et al., 2009; Lee and Lee, 2014). The activation of these signaling pathways increases the production of pro-inflammatory cytokines, endothelial adhesion molecules, and chemotactic mediators that promote the infiltration of monocytes in AT and the differentiation into pro-inflammatory M1 macrophages (Shoelson et al., 2006). Infiltrating macrophages produce and secrete many inflammatory mediators that promote local and systemic pro-inflammatory status and impair insulin signaling (Haase et al., 2014).

The effects of these cytokines are mediated by stimulation of IκB kinase β (*IKK*β) and *JNK1*, expressed in myeloid and insulin-targeted cells (McLaughlin et al., 2016).

JNK is one of the most investigated signal transducers in obesity models of IR. It is activated after exposure to many inflammatory stimuli including cytokines, free fatty acids, and activation of cellular pathways, such as UPR (Aguirre et al., 2000; Ozcan et al., 2004). Once activated, JNK starts a pro-inflammatory gene transcription and inhibits insulin signaling pathway through inhibitory serine–threonine phosphorylation of IRS-1, thereby decreasing PI3K/PKB signaling (Tanti et al., 1994; Gual et al., 2005). In obese mice (ob/ob and diet-induced obesity), JNK activity is increased in AT compared to control mice. The role of *Jnk1* in adipocytes has been investigated using tissue-specific *Jnk1*-deficient mice. These mice are protected against the development of IR when fed a HFD. This effect is tissue specific because *Jnk1* deficiency in adipocytes does not affect muscle insulin sensitivity (Hirosumi et al., 2002; Sabio et al., 2008).

Obesity is also associated with the activation of NF-κB inflammatory pathway. In physiological conditions, NF-κB proteins are retained in the cytoplasm of myeloid and insulin-targeted cells by a family of inhibitors called inhibitors of κB (IκBs) (McLaughlin et al., 2017). Activation of IKK kinase complex (that contains IKKα and IKKβ subunits) induces proteasomal degradation of IκBα, leading to NF-κB nuclear translocation. This culminates in the increased expression of several NF-κB target genes [e.g., *IL-6*, *TNF*α, interferon-γ (*IFN-*γ), transforming growth factor-β (*TGF-*β), monocyte chemotactic protein-1 (*MCP-1*), and interleukin-1β (*IL-1*β)], which further exacerbate IR progression (Shoelson et al., 2006; Panahi et al., 2018). IKKβ deficiency in adipocytes totally prevents the expression of *IL-6* and *TNF-*α induced by free fatty acid, while its activation inhibits the expression of anti-inflammatory cytokines such as adiponectin and leptin (Jiao et al., 2011). Therapeutic approaches capable of targeting these pathways and improving insulin sensitivity in obese subjects will be further discussed below in this review.

Macrophages represent another important cell type in mediating the obesity-induced inflammation in the AT. During obesity, macrophages infiltrate the AT and secrete many pro-inflammatory cytokines (Weisberg et al., 2003). These mediators have local effects on adipocytes and resident immune cells (e.g., neutrophils, B cells, and T cells) and circulate in the periphery, where they affect the liver and skeletal muscle insulin sensitivity (Weisberg et al., 2003; Xu et al., 2003; Haase et al., 2014; McLaughlin et al., 2017).

Myeloid cells activate another molecular pathway, called inflammasome, in obesity (Lee and Lee, 2014). Macrophages and other innate immune cells may trigger inflammatory responses by detecting pathogen- or danger-associated molecular patterns (PAMPs or DAMPs) using a broad variety of pattern-recognition receptors such as TLRs and NLRs (Pedra et al., 2009; Vandanmagsar et al., 2011). Compelling evidence shows that NLRP3 (the most studied member of the NLR family) activation by DAMPs (generated by nutrient excess in obesity) plays a key role behind the chronic inflammation characteristic of obesity and IR (Stienstra et al., 2010, 2011, 2012; Zhou et al., 2010; Koenen et al., 2011; Vandanmagsar et al., 2011; Lee et al., 2013).

NLRP3 is present in several tissues and cell types (Vandanmagsar et al., 2011). It is unclear which cell compartments in AT express the inflammasome components; however, immunostaining of AT sections of obese mice confirmed a strong co-localization of NLRP3 with macrophage marker F4/80 in crown-like structures (Vandanmagsar et al., 2011). Once activated, NLRP3 interacts with procaspase-1 through an adaptive protein forming the NLRP3 inflammasome (Schroder et al., 2010; Davis et al., 2011; Shao et al., 2015). This results in the processing and activation of caspase-1, which mediates the maturation and secretion of IL-1β and IL-18 by macrophages (Shoelson et al., 2003; Schroder et al., 2010; Davis et al., 2011; Shao et al., 2015). The primary role played by NLRP3 inflammasome is also supported by evidence that genetic ablation of *NLRP3*^–/–^ prevents the obesity-induced inflammasome activation in AT and protects against HFD-induced IR (Vandanmagsar et al., 2011). Caloric and exercise-mediated weight loss in obese people with T2D reduces *NLPR3* and *IL-1*β gene expression in abdominal subcutaneous AT and improves systemic insulin sensitivity (Vandanmagsar et al., 2011).

Inflammasome-activated IL-1β is a major cytokine produced by macrophages (Sims and Smith, 2010). Its enhanced production in pancreatic islets and insulin-sensitive tissues is associated to T2D (Hotamisligil et al., 1993; Donath and Shoelson, 2011). In obesity, chronic rise in circulating nutrients such as glucose and free fatty acids (FFAs) resulted in over-expression of IL-1β in pancreatic β-cells (Maedler et al., 2002; Böni-Schnetzler et al., 2008; Fei et al., 2008; Böni-Schnetzler et al., 2009). It is now clear that IL-1β is a key cytokine in the etiology of T2D since it has been implicated in IR, β-cell dysfunction, and death (Eizirik and Mandrup-Poulsen, 2001; Donath et al., 2008, 2019). IL-1β alters the insulin sensitivity of AT by suppressing insulin signaling; exposure to IL-1β of murine and human adipocytes decreases insulin-stimulated glucose uptake and lipogenesis (Lagathu et al., 2006; Jager et al., 2007; Fève and Bastard, 2009), reduces glucose transporter type 4 (GLUT4) expression, and inhibits GLUT4 translocation to the plasma membrane (Jager et al., 2007; Ballak et al., 2015).

The pro-apoptotic effects of IL-1β on β-cells derive from a complex network of signaling events triggered by IL-1β binding to its cognate receptor, whose expression is higher in β-cells than in other tissues (Böni-Schnetzler et al., 2009). Once cytokine binds its receptor, the co-receptor is recruited, and this results in the formation of the heterodimer receptor transmembrane complex. Both receptors and co-receptors share a cytoplasmic motif, the Toll/IL-1 receptor (IL-1R) domain, which is required to initiate intracellular signaling by recruitment of different adaptor proteins and kinases, including the myeloid primary response differentiation-88 protein and the interleukin-1-associated kinase receptor. This leads to activation of MAPK and NF-κB signaling pathways. The activation of these two signaling pathways causes variations in gene expression, therefore triggering the apoptotic cell death program in β-cells (Donath et al., 2008, 2019). The pro-apoptotic effects mediated by NF-kB depend on the cell type, nature, and duration of the stimulus. Indeed, NF-kB activation in β-cells is more marked, rapid, and sustained than in other cell types (Ortis et al., 2006). MAPKs also take part in β-cell apoptosis through transcription-independent mechanisms, such as regulating B-cell lymphoma 2 protein activity (Donath et al., 2008, 2019). The combined use of IFN-γ and IL-1β induces the activation of an additional mechanism, the so-called non-canonical NF-kB pathway, also implicated in the pro-apoptotic effects of IL-1β on β-cells (Meyerovich et al., 2016).

IL-1β is also implicated in cardiovascular and microvascular long-term complications (nephropathy, retinopathy, and polyneuropathy) of diabetes (Herder et al., 2013, 2015; Agrawal and Kant, 2014; Stahel et al., 2016; Donath et al., 2019). Endothelial cell damage is a crucial and an early manifestation of diabetic-associated vascular complications (van den Oever et al., 2010; Gilbert, 2013; Liu et al., 2014). Among the multiple and potential mechanisms that contribute to this phenomenon, a crucial role is played by chronic low-grade inflammation. IL-1β has been reported to cause endothelial cell damage in isolated mesenteric rat micro-vessels (Vila and Salaices, 2005; Shashkin et al., 2006). Vallejo et al. (2014) have shown that the deleterious effects of IL-1β on endothelial cells are due to the IL-1R-mediated activation of NADPH oxidase, which stimulates the production of superoxide anion (Vallejo et al., 2014). Over-activation of NADPH oxidase has also been associated with excess ROS production and the development of atherosclerosis in diabetic vasculopathy (Olukman et al., 2010; Gray et al., 2013).

IL-18 is another pro-inflammatory mediator activated by inflammasome and produced and released by human AT (Wood et al., 2005). IL-18 plasma levels are increased in obese people and in individuals with T2D (Moriwaki et al., 2003; Evans et al., 2007) while being restored in subjects who have lost weight following bariatric surgery (Schernthaner et al., 2006). It is a powerful pro-inflammatory cytokine that increases the maturation of T and NKs, as well as the production of other pro-inflammatory cytokines, exacerbating the obesity-induced systemic inflammation (Weisberg et al., 2003).

Likewise, IL-6 has been suggested to be involved in the development of obesity-related and T2D-related IR (Fève and Bastard, 2009). IL-6 leads to impaired insulin signaling, and this occurs primarily by inhibition of insulin-stimulated tyrosine phosphorylation of IRSs both in the liver and in AT (Senn et al., 2002; Klover et al., 2003; Lagathu et al., 2003; Rotter-Sopasakis et al., 2004; Fève and Bastard, 2009). Nonetheless, conflicting results have been reported for IL-6 action on skeletal muscle (Fève and Bastard, 2009). Carey et al. (2006) have shown that IL-6 increases GLUT4 translocation on plasma membrane and promotes insulin-stimulated glucose uptake in myotubes (Carey et al., 2006). Nevertheless, it has also been shown that in murine skeletal muscle cells, IL-6 is capable of reducing insulin-stimulated glucose uptake through JNK activation (Nieto-Vazquez et al., 2008). In pancreas, IL-6 impairs insulin secretion and has pro-apoptotic effects on β-cells (Ellingsgaard et al., 2008). An opposite effect is carried out on α-cells; IL-6 prevents α-cells apoptosis and induces the secretion of glucagon-like peptide-1. This could be considered an adaptive mechanism to compensate for β-cell failure (Ellingsgaard et al., 2008, 2011; Akbari and Hassan-Zadeh, 2018). Such findings support the tissue-specific effect of IL-6 on glucose homeostasis, which depends on several factors, such as concentrations, targets, and signaling pathways activated.

The IL-6 signaling cascade involves activation of the Janus kinase (JAK)-signal transducer and activator of transcription (STAT) pathway (Fève and Bastard, 2009; Dodington et al., 2018). It serves as a crucial downstream mediator for a variety of hormones, cytokines (Gadina et al., 2018), and growth factors, including growth hormone, leptin, IL-6, and IFN-γ (Dodington et al., 2018). There are four identified members in the JAK kinase family (JAKs 1-3 and Tyk2), which associate with cytokine and growth factor receptors. JAK-mediated signaling leads to the activation of seven STAT family members (STATs 1-4, 5A, 5B, and 6). STAT proteins have cell- and tissue-specific distribution that influences their specificity and function (Schindler and Darnell, 1995; Richard and Stephens, 2011, 2014). The regulation of tissue-specific genes and the ability to have cell-specific tasks appear to be important physiological roles of the JAK/STAT pathway (Richard and Stephens, 2011). JAK/STAT signaling in the peripheral metabolic organs modulates a multitude of metabolic processes, including adiposity, energy expenditure, glucose tolerance, and insulin sensitivity (Dodington et al., 2018). This signaling pathway mediates the action of several hormones that have profound effects on adipocyte development and function. Adipocytes also produce hormones that utilize this pathway (Richard and Stephens, 2011). The expression of several STATs is modulated during adipogenesis (Richard and Stephens, 2011). Additional functions of JAK/STAT signaling in adipocytes include the transcriptional regulation of genes involved in insulin action and lipid and glucose metabolism (Richard and Stephens, 2011). JAK2, STAT3, and STAT5 are essential for signaling through both the growth hormone and leptin receptors and have been characterized in WAT (Dodington et al., 2018). As the major upstream kinases required for STAT activity, it is not surprising that JAK proteins also play important roles in the control of AT function (Gurzov et al., 2016). Adipose-specific *Jak2* KO mice have demonstrated defective lipolysis, increased body weight and adiposity compared to controls, leading to IR (Nordstrom et al., 2013; Shi et al., 2014; Corbit et al., 2017). Similarly, loss of either *Stat3* or *Stat5* in AT contributes to increased weight gain, adiposity, and impaired lipolysis (Dodington et al., 2018). There is a controversy over the effects of adipocyte JAK2/STAT5 on insulin sensitivity. Some studies have shown IR (Shi et al., 2014) while others have demonstrated enhanced whole-body insulin sensitivity in the absence of JAK2 or STAT5 (Nordstrom et al., 2013; Corbit et al., 2017). This inconsistency might be due to a variety of factors including tissue specificity and cell stage-dependent expression of the *cre* transgene, mouse genetic background, physiologic status, and other environmental factors in which the experiments were performed (Dodington et al., 2018). Although the direct role of STAT1 in the anti-adipogenic action of IFN-γ was not investigated, experiments using pharmacological inhibitors show that the JAK-STAT1 pathway plays a key role in the ability of IFN-γ to induce IR, decline triglyceride stores, and down-regulate expression of lipogenic genes in mature human adipocytes (Richard and Stephens, 2014). The increased IFN-γ levels and JAK-STAT1 signaling in obesity contribute to AT dysfunction and IR (Gurzov et al., 2016).

Emerging evidence demonstrates that the highly conserved and potent JAK/STAT signaling pathway is dysregulated in metabolic diseases, including obesity and T2D (Gurzov et al., 2016; Dodington et al., 2018). Studies show that many STAT activators play an important role in the regulation of adipocyte gene expression and exhibit differential expression in the condition of obesity and/or IR (Richard and Stephens, 2014). Obesity increases levels of IL-6 in WAT that, in turn, chronically activate intracellular JAK-STAT3 signaling. Chronic JAK-STAT3 signaling induced by IL-6 leads to the increased expression of suppressor of cytokine signaling-3 that not only negatively regulates IL-6 signaling but also hinders insulin action, eventually resulting in obesity and IR (Wunderlich et al., 2013). JAK/STAT signaling can have both physiological and pathological roles depending on the context. It is difficult to speculate how JAK/STAT inhibition will affect individuals with obesity and diabetes (Dodington et al., 2018). This complexity highlights the need for validation of the relative contribution of STAT proteins in human samples. Further studies will also be required to reveal the complex roles of the JAK-STAT pathway in adipocytes, obesity, and IR. Manipulation of this pathway within AT is a novel therapeutic approach for the treatment of obesity and diabetes.

Systemic inflammation is characterized by high circulating levels of inflammatory mediators and immune cells that infiltrate insulin-dependent tissues (Weisberg et al., 2003). As has already been discussed in the review, WAT is the main site where low-grade systemic inflammation begins (Weisberg et al., 2003; Xu et al., 2003). Accumulation of lipids that occurs in AT during obesity triggers an inflammatory response that results in an increased secretion of several inflammatory cytokines (Haase et al., 2014; Raciti et al., 2017). Such molecules can also activate JNK and NF-κB signaling pathways in the liver and skeletal muscle, thus inhibiting systemic insulin signaling (Hotamisligil et al., 1993; Ciccarelli et al., 2016).

**FIGURE 1:**
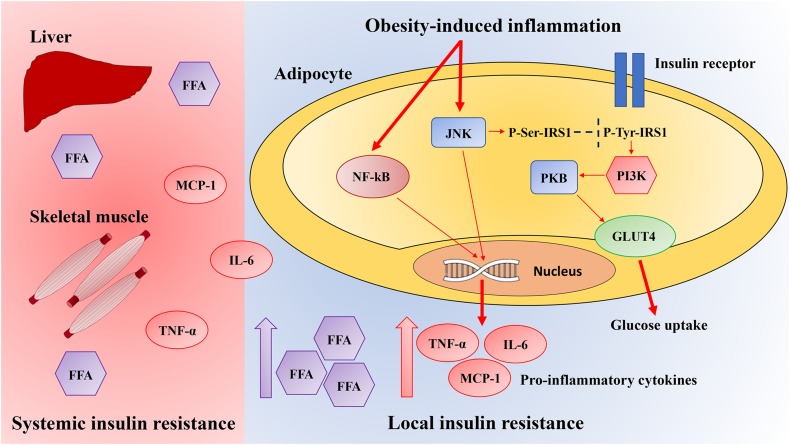
Pathways linking local obesity-induced inflammation to systemic insulin resistance. Obesity results in the activation of the inflammatory signaling pathways mediated by JNK and nuclear factor-kappa B (NF-κB). Once activated, these pathways induce the production of several pro-inflammatory cytokines in adipocytes, which contribute to insulin resistance and pro-inflammatory macrophages infiltration. Activation of the JNK signaling pathway starts the transcription of pro-inflammatory genes and inhibits the insulin signaling pathway through the insulin receptor substrate-1 (IRS-1) inhibitory serine phosphorylation, which reduces the phosphatidylinositol 3-kinase (PI3K)/protein kinase B (PKB) signaling pathway. Instead, NF-κB signaling pathway activation culminates in the increased expression of several NF-κB target genes such as tumor necrosis factor-α (TNF-α), interleukin-6 (IL-6), and monocyte chemotactic protein-1 (MCP-1), which leads to serine phosphorylation of IRS-1, therefore preventing insulin signaling. The inflammatory mediators including free fatty acids (FFA), IL-6, TNF-α, and MCP-1 also spread through systemic circulation and activate JNK and NF-κB signaling pathways in the liver and skeletal muscle, inhibiting systemic insulin signaling. GLUT4, glucose transporter type 4.

Obesity-induced inflammation initiates in WAT and then spreads to other tissues, resulting in low-grade systemic inflammation. In obesity, both liver and skeletal muscle exhibit signs of local inflammation (Figure 1).

Skeletal muscle is the principal organ for insulin-stimulated glucose uptake (i.e., capable for 80% of glucose disposal in human), and muscle IR plays a key part in T2D etiology (DeFronzo and Tripathy, 2009; Honka et al., 2018). Obesity contributes to the development of chronic muscle inflammation, characterized by increased pro-inflammatory M1 macrophage infiltration (Fink et al., 2013, 2014). These macrophages secrete many cytokines, which have been shown to trigger inflammatory pathways within myocyte, culminating in decreased insulin signaling (Varma et al., 2009; Pillon et al., 2012; Patsouris et al., 2014).

In the liver, obesity leads to increased infiltration and pro-inflammatory activation of two major macrophage groups: Kupffer cells (i.e., resident specialized hepatic macrophages) and monocyte-derived recruited hepatic macrophages (Tencerova et al., 2015). Further work indicates that, during obesity, the number of Kupffer cells remains unaffected, whereas the accumulation of monocyte-derived recruited macrophages increases several times (Morinaga et al., 2015). It has been demonstrated that ATM-released inflammatory mediators lead to hepatic IR by reducing insulin signaling (Morinaga et al., 2015). These inflammatory mediators also contribute to liver steatosis by promoting lipogenesis and toxic ceramide biosynthesis (Schubert et al., 2000; Obstfeld et al., 2010).

## Role of Innate and Adaptive Immunity in Obesity

Recently, as specified above, particular attention has been paid to the role played by macrophages in AT inflammation. Nevertheless, many other immune cells (both innate and adaptive immune systems) are involved in the development of local and systemic inflammation and IR.

### Macrophages

During obesity, different types of both innate and adaptive immune cells accumulate in AT (Lackey and Olefsky, 2016). Macrophages are the most abundant and constitute up to 40% of all AT cells in obesity (Lumeng et al., 2007a; Lee et al., 2018). They are suggested to be the major source of pro-inflammatory cytokines (Samuel and Shulman, 2012), which can cause IR (Xu et al., 2003).

Obesity is associated with the recruitment of M1-polarized macrophages, which secrete pro-inflammatory cytokines such as TNF-α and IL-1β (Han and Levings, 2013). Inflamed AT is characterized by the combination of an increase in total macrophages and an increased ratio of M1 to M2 (anti-inflammatory) macrophages, which comes along obesity and is linked with the development of IR (Lumeng et al., 2007a; Reilly and Saltiel, 2017). However, we should consider that macrophage inflammation in response to obesity is not identical to the classic M1 activation state observed in inflammation associated with acute infection. As ATMs express a different set of surface markers, the pro-inflammatory activation in the setting of obesity has been referred to as metabolic activation, or Me, rather than M1 (Reilly and Saltiel, 2017). The pro-inflammatory macrophages in obese AT also upregulate the expression of genes that encode proteins involved in lipid metabolism. Hence, they can be also distinguished from classically activated macrophages (Xu et al., 2013; Henao-Mejia et al., 2014; Reilly and Saltiel, 2017).

### Neutrophils

While ATMs are the pivotal effector innate immune cells causing IR, alterations in several other innate immune cell types in obese AT contribute to the initiation and/or progression of AT inflammation (Lee et al., 2018).

Neutrophils are the leukocyte subpopulation (Chmelar et al., 2013) and granulocytes involved in innate immunity (Asghar and Sheikh, 2017; Kane and Lynch, 2019). They comprise up to 90% of all granulocytes in the blood but are relatively rare in AT of lean mice (Talukdar et al., 2012; Kane and Lynch, 2019). However, neutrophils are among the first responders recruited to AT in mice as early as 3 days after the initiation of HFD. Talukdar et al. (2012) reported that the early recruitment of neutrophils was prolonged over 90 days on HFD (Talukdar et al., 2012; Chmelar et al., 2013). We should take into account that another study already showed that this migration is transient (Elgazar-Carmon et al., 2008).

Neutrophils stimulate AT inflammation by producing TNF-α and MCP-1 (Dam et al., 2016; Trim et al., 2018). Neutrophils also produce elastase, which impairs glucose uptake in AT (Wang et al., 2009) and promotes IR by degrading IRS-1 (Talukdar et al., 2012; McLaughlin et al., 2017; Lee et al., 2018). The activity of elastase is also increased in the AT of HFD mice, corresponding to the number of infiltrated neutrophils (Talukdar et al., 2012; Chmelar et al., 2013). Genetic deletion of elastase attenuates macrophage influx into the AT of obese mice and results in improved insulin sensitivity (Talukdar et al., 2012; Hotamisligil, 2017).

### Dendritic Cells

Dendritic cells are specialized antigen-presenting cells that link the innate and adaptive immunity (Bertola et al., 2012; McLaughlin et al., 2017; Chung et al., 2018) by presenting antigens to T cell receptors (Steinman, 2008).

Dendritic cells accumulate in AT of mice fed an HFD and in the subcutaneous AT of obese humans (Cho et al., 2016). They likely induce the pro-inflammatory microenvironment through macrophage recruitment and IL-6 production (Stefanovic-Racic et al., 2012). Blocking their accumulation improves insulin sensitivity in obese mice (Cho et al., 2016).

DCs inhibit healthy expansion of AT, and depletion of these cells improves glucose homeostasis in mice (Hotamisligil, 2017). Adipose-recruited DCs have been shown to be associated with the deregulation of chemerin, a particular adipokine (Ghosh et al., 2016; Lu et al., 2019).

Altogether, these studies suggest a pathogenic role for DCs in the development of obesity in mice and humans. Mice with deletion of Fms-like tyrosine kinase 3 ligand (*Flt3L*), that lack DCs, revealed reduced macrophage number in the AT and liver as well as improved insulin sensitivity in diet-induced obesity. Administration of recombinant Flt3L to these mice reversed this phenotype (Chung et al., 2018).

### Mast Cells

Mast cells are innate immune cells (Liu et al., 2009) that originate from CD34^+^, CD13^+^, and CD17^+^ multipotent hematopoietic stem cells (Zelechowska et al., 2018). AT is a reservoir of mast cells (Zelechowska et al., 2018). There is a significant increase in the number of mast cells in the WAT of mice and humans with obesity (Liu et al., 2009) and/or T2D (Lackey and Olefsky, 2016).

Mast cells promote AT low-grade inflammation in obesity (Liu et al., 2009). They mediate the macrophage infiltration (Liu et al., 2009). Interestingly, mast cells are regulated by IL-6 and IFN-γ but not via TNF-α (Liu et al., 2009; Sun et al., 2012). IL-6 and IFN-γ play a crucial role in the ability of mast cells to regulate metabolism, and they may mediate diet-induced obesity and diabetes (Zelechowska et al., 2018). Immature mast cells that infiltrate into AT the non-obese stage progressively mature and promote obesity and diabetes progression (Hirai et al., 2014).

Mast cells tend to degranulate (Zelechowska et al., 2018), resulting in the secretion of a large amount of pro-inflammatory and immunomodulatory mediators, such as histamine, cytokines, and chemokines (Sun et al., 2012; Zelechowska et al., 2018). Hence, they have a key role in allergic responses and AT homeostasis (Sun et al., 2012). Mast cell deficiency is associated with improved insulin sensitivity (McLaughlin et al., 2017).

### B Cells

Lymphocytes account for up to 10% of non-adipocytes cells in human AT and include T cells, B cells, NKs, NKTs, and ILC2s (McLaughlin et al., 2017).

B cells are an important component of the adaptive immunity that release immunoglobulins or antibodies to recognize the cognate antigen. This feature differs from the cell-mediated immunity where T cells recognize processed antigenic peptides presented by antigen-presenting cells (Sun et al., 2012). B cells in AT are phenotypically different from B cells found in other tissues, as B cells in AT have unique genetic markers (Dam et al., 2016). They are present across all known AT depots but are less well characterized than T cells (Kane and Lynch, 2019).

B cells have also been shown to be pathogenic in obesity (Kane and Lynch, 2019). B cells accumulate in the AT of obese mice relative to lean mice and become more inflammatory, producing chemokines that promote the recruitment of neutrophils, T cells, and monocytes (Kane and Lynch, 2019). B cells promote pro-inflammatory activation of ATMs and T cells (Winer et al., 2011; Lee et al., 2018).

B cells modulate IR by accumulating in the AT of obese mice (Winer et al., 2009). It has been reported that B cell accumulation precedes T cell accumulation during the development of obesity (Lau et al., 2012). B cells might contribute to AT inflammation by producing immunoglobulin G antibodies or pro-inflammatory cytokines. The B cells from obese mice release a more inflammatory repertoire of cytokines (Michelsen et al., 2004; Winer et al., 2011; Ding et al., 2012).

Obese mice with B cell deficiency reduce IR (DeFuria et al., 2013; Hotamisligil, 2017). Transfer of B cells from obese donor mice causes impaired insulin sensitivity and glucose homeostasis in the recipients (Winer et al., 2011; Hotamisligil, 2017). By contrast, there are also tolerance-promoting B regulatory cells in AT, and their number is decreased in models of obesity (Nishimura et al., 2013; Hotamisligil, 2017).

### T Cells

CD3^+^ T cells constitute the largest AT immune-cell population next to macrophages, and their abundance is increased in HFD obese mice (Lee et al., 2018). T cells can be divided into two subtypes depending on the markers on their surface, CD4 and CD8 T cells (Pennock et al., 2013).

Obesity is associated with an increase in the number of CD8^+^ T cells in AT (Henao-Mejia et al., 2014), and these cells promote macrophage differentiation and chemotaxis (Nishimura et al., 2009).

CD4^+^ T cells identify major histocompatibility complex class II, presented on the surface of antigen-presenting cells like DCs, macrophages, and B cells (Dam et al., 2016; Trim et al., 2018). CD4^+^ T cells are subclassified into pro-inflammatory T helper 1 (Th1) and Th17 cells, anti-inflammatory Th2 cells, and T regulatory cells (Tregs). The number of CD3^+^CD4^+^ Th1 cells is increased in obesity, and they stimulate AT inflammation by secreting IFN-γ. In contrast, the number of CD3^+^CD4^+^ Th2 cells is declined in obese AT (Winer et al., 2009; Lee et al., 2018).

Treg cells (CD3^+^CD4^+^FOXP3^+^) are a small subset of CD4^+^ T lymphocytes that inhibit inappropriate inflammation. Treg population in lean AT is characterized by high peroxisome proliferator-activated receptor γ (*PPAR*γ) expression. These Tregs play a critical role in maintaining AT inflammatory tone and insulin sensitivity (Lee et al., 2018). The decline in the numbers of AT Treg cells during obesity contributes to increased AT inflammation (Zhou et al., 2010; Henao-Mejia et al., 2014) and IR (Feuerer et al., 2009; Lee et al., 2018).

Invariant natural killer T cells (iNKTs) are lipid-antigen-reactive T cells restricted by the major histocompatibility complex-like molecule CD1d (Lee et al., 2018). iNKT cells form a subset of lymphocytes in normal AT. The number of iNKTs is reduced in obesity (Lee et al., 2018). Furthermore, mice lacking iNKTs shows increased weight gain, larger adipocytes, and IR on HFD. This is associated with increased infiltration of macrophages into AT (Lynch et al., 2012).

Collectively, the network of T and B cells has crucial effects to influence macrophage infiltration. Thus, pro-inflammatory macrophages are the final effector cells that induce IR (Lee et al., 2018).

## Obesity-Induced at Inflammation Triggers

**FIGURE 2:**
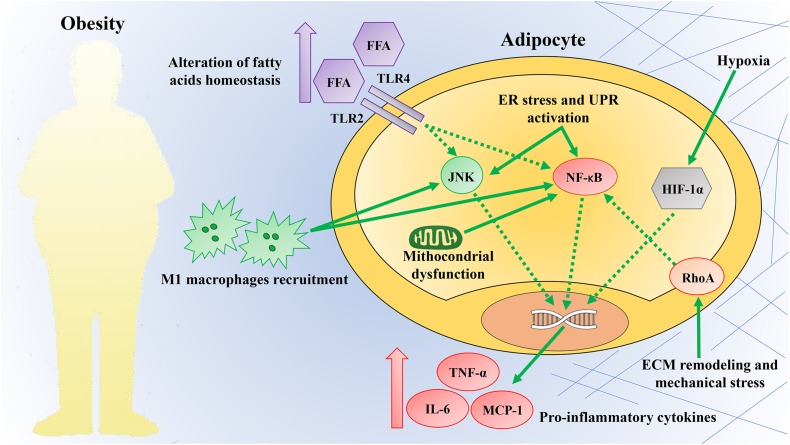
Obesity triggers inflammation. Obesity provides a plethora of intrinsic and extrinsic signals capable of triggering an inflammatory response in AT. These mechanisms are commonly considered the link between chronic caloric excess and adipose tissue inflammation. Some of these mechanisms include dysregulation of fatty acid homeostasis, increased adipose cell size and death, local hypoxia, mitochondrial dysfunction, endoplasmic reticulum (ER), and mechanical stress. These triggers converge on the activation of the c-Jun N-terminal kinase (JNK) and nuclear factor-kappa B (NF-κB) pathways, commonly considered signaling hubs. The activation of these pathways increases the production of pro-inflammatory cytokines and promotes the infiltration of pro-inflammatory M1 macrophages. TLR2, Toll like receptor 2; TLR4, Toll like receptor 4; FFA, free fatty acids; UPR, unfolded protein response; HIF-1α, hypoxia-inducible factor-1α; RhoA, ras homolog gene family, member A; TNF-α, tumor necrosis factor-α; IL-6, interleukin-6; MCP-1, monocyte chemotactic protein-1; ECM, extracellular matrix.

There is a limited understanding of how obesity-induced inflammation in AT is triggered. However, potential mechanisms identified include dysregulation of fatty acids homeostasis, increased adipose cell size and death, local hypoxia, mitochondrial dysfunction, ER stress, and mechanical stress (Figure 2) (Heilbronn and Liu, 2014; Reilly and Saltiel, 2017). These mechanisms are recognized as the link between chronic caloric excess and AT inflammation or as factors that may perpetuate chronic tissue inflammation (Burhans et al., 2018). The list of potential mechanical links mentioned here is not complete, and it is likely that the triggers leading to AT inflammation have not yet been identified.

### Dysregulated Fatty Acids Homeostasis

Saturated fatty acids promote inflammatory activation of macrophages, partially mediated by indirect binding to TLR4 and TLR2 (Konner and Bruning, 2011), resulting in the activation of NF-κB and JNK pathways (Shi et al., 2006; Milanski et al., 2009).

Once these pathways have been stimulated, many chemokines (e.g., MCP-1 and TNF-α) are produced and released from adipocytes, resulting in inflammatory macrophage infiltration (Reilly and Saltiel, 2017). In obesity, in addition to an increased intake of saturated fatty acids, *TLR4* and *TLR2* expression are increased in the AT, further supporting the role of these receptors in obesity-associated inflammatory signaling (Husam et al., 2008; Vitseva et al., 2008). In regard to this, acute lipid infusion is enough to stimulate AT inflammation and systemic IR in wild-type mice, and these effects are prevented in *TLR4*-deficient mice (Shi et al., 2006). Based on these findings, TLR4 appears to be an interesting candidate for linking dietary fatty acids with AT inflammation and IR (Poggi et al., 2007). Despite saturated fatty acids, unsaturated omega-3 and -9 fatty acids have beneficial effects and alleviate AT inflammation (Oliveira et al., 2015).

### Adipocyte Hypertrophy, Hypoxia, and Death

WAT plays a major role in regulating systemic energy homeostasis, which acts as a safe reservoir for fat storage. In response to changes in nutritional status, AT expands by increasing the number (hyperplasia) and size of the adipocytes (hypertrophy) (Sun et al., 2011; Longo et al., 2018; Hammarstedt et al., 2018). Cross-sectional studies have demonstrated that the size of visceral adipocytes is negatively correlated with insulin sensitivity (O’Connell et al., 2010; Hardy et al., 2011), and these findings allow proposing adipocyte size as an IR determinant (O’Connell et al., 2010).

Thus, the evidence indicating that adipocyte hypertrophy certainly contributes to AT inflammation is quite convincing at the present. Increased adipocyte size is characterized by a higher rate of adipocyte death and macrophage recruitment. Larger adipocytes exhibit an altered chemoattractant and immune-related proteins secretion that may promote pro-inflammatory macrophage infiltration (Jernas et al., 2006; Heilbronn and Liu, 2014). Most of these infiltrated macrophages surround necrotic adipocytes and form crown-like structures. In obese rodents as well as humans, necrosis-related factors further attract monocytes in AT where they uptake the lipids released by dead adipocytes (Cinti et al., 2005; Murano et al., 2008; Choe et al., 2016). As described above, the recruited monocytes have a pro-inflammatory phenotype and secrete cytokines and reactive oxygen species in neighboring adipocytes that interfere with insulin signaling (Shapiro et al., 2011). An increase in the number of dead adipocytes has been recognized to prevent normal AT functions and cause inflammation (Choe et al., 2016).

During adipocyte hypertrophy, angiogenesis is initiated to supply oxygen to the expanding tissue. If the AT expansion is very rapid, the vasculature cannot fulfill the oxygen requirement and hypoxia occurs (Gealekman et al., 2011; Sun et al., 2011; Trayhurn, 2013).

Hypoxia is a strong metabolic stressor. Current evidence reveals that hypoxia develops as AT expands because of a relative under perfusion of the enlarged AT or increased oxygen utilization (Gealekman et al., 2011; Sun et al., 2011; Trayhurn, 2013; Lee et al., 2014).

Cellular hypoxia can initiate inflammation by activating hypoxia-inducible factor-1 (*HIF-1*) gene program. Activated HIF-1α translocates to the nucleus where it recognizes and binds the HREs on DNA. The binding to HREs promotes not only the expression of many genes involved in the angiogenesis but also inflammation (Trayhurn, 2013; Fiory et al., 2019). These include vascular endothelial growth factor, insulin-like growth factor 2, transforming growth factor α, as well as nuclear factor of kappa light polypeptide gene enhancer in B-cells 1 and inflammatory cytokines such as interleukin-33 and 18 (Shi and Fang, 2004). It has been shown that adipocyte-specific *HIF-1*α deletion prevents obesity-induced inflammation and IR (Lee et al., 2014).

### Mitochondrial Dysfunction

Mitochondria are present in almost all eukaryotic cells and are responsible for cellular energy production, calcium signaling, and apoptosis (Osellame et al., 2012). Alterations in mitochondrial functions are capable of causing inflammation, oxidative stress, cell death, and metabolic dysfunction (Hock and Kralli, 2009; Kim et al., 2016). A number of studies in obese mice and human subjects have shown that mitochondrial dysfunction is strongly associated with pathological conditions such as inflammation, IR, and T2D (Silva et al., 2000; Petersen et al., 2003, 2004; Morino et al., 2005; Woo et al., 2019). Alterations in mitochondrial functions and reductions in mitochondrial DNA content have been reported in obese (ob/ob) and diabetic (db/db) mice (Choo et al., 2006; Koh et al., 2007). A comparable decrease in mitochondrial activity has also been observed in human AT from obese individuals (Yin et al., 2014).

The mitochondrial dysfunction leads to inflammation through modulating redox-sensitive inflammatory mechanisms such as NF-κB or direct inflammasome activation (Vaamonde-García et al., 2012; López-Armada et al., 2013). The activation of both pathways induces an upregulation of inflammatory cytokines and adhesion molecules secretion, resulting in a substantial amplification of the inflammatory response (Escames et al., 2011; López-Armada et al., 2013).

Petersen et al. (2003, 2004) reported that the mitochondrial dysfunction also contributes to ectopic fat accumulation (i.e., accumulation of intracellular fatty acid metabolites such as fatty acyl-CoA and diacylglycerols), which blocks insulin signaling.

Woo et al. (2019) suggest a new hypothesis that adipocyte mitochondrial dysfunction causes AT inflammation and systemic IR by inducing fatty acids accumulation in adipocytes, and resulting in adipocyte hypertrophy and hypoxia.

### ER Stress

ER is a cellular organelle that exhibits high sensitivity to cellular nutrients and energy status (Hummasti and Hotamisligil, 2010). Many genetic and environmental hits can alter the functions of ER and therefore contribute to ER stress (Hummasti and Hotamisligil, 2010). Several studies have shown that the incorrect functioning of the UPR (i.e., ER-stress mitigation system in eukaryotes) is associated with chronic metabolic diseases including obesity, IR, and T2D (Ozcan et al., 2006; Boden et al., 2008; Sharma et al., 2008; Gregor et al., 2009). It has been shown in mice that obesity results in increased ER stress, particularly in the liver and AT. Indeed, the expression of most ER stress markers and chaperones is strongly BMI-related and associated with AT insulin sensitivity (Sharma et al., 2008). Additionally, a weight-loss gastric bypass surgery has been shown to enhance insulin sensitivity and decrease ER stress in obese (Gregor et al., 2009).

Inflammation is the predominant mechanism by which ER stress negatively affects metabolic homeostasis. The primary mechanisms by which ER stress establishes inflammatory mechanisms in AT involve the activation of NF-κB, JNK, and apoptosis signaling pathways.

In response to ER stress, the three UPR branches are activated. The activation of two branches is mediated by protein kinase RNA (PKR)-like ER kinase (PERK) and activating transcription factor 6 (ATF6). This activation stimulates NF-κB signaling pathway, resulting in the subsequent inhibition of insulin action via IRS-1 phosphorylation. In addition, the branch mediated by inositol-requiring enzyme 1 results in the activation of the JNK signaling pathway (Hotamisligil, 2010; Hummasti and Hotamisligil, 2010). There is also crosstalk between the three branches. For example, spliced X-box binding protein 1, as well as activating transcription factor 4, induces the production of the inflammatory cytokines IL-6, interleukin-8, and MCP-1 by human endothelial cells (Hotamisligil, 2010).

A further important function of UPR is to activate pro-apoptotic signaling pathways in order to prevent the release and accumulation of misfolded proteins, which may have adverse effects on cellular functions (Hotamisligil, 2010). However, ER stress-induced apoptosis may also contribute to increased inflammatory signaling and other aspects of metabolic diseases. For instance, adipocyte death in obesity has been suggested as a potential trigger for the recruitment of macrophages and other inflammatory cells (Cinti et al., 2005), as described in the review. Evidence also indicates that ER stress is essential for β-cell development and survival (Harding et al., 2001; Scheuner et al., 2001; Zhang et al., 2002).

In 2016, we have reported that UPR hyper-activation by glucose insult leads to a pro-inflammatory phenotype in preadipocytes. Cells exposed to hyperglycemia release an increased amount of pro-inflammatory cytokines, chemokines and IL-12 lymphokine, which can trigger inflammation by affecting inflammatory cells. However, such effects are prevented by a chemical chaperone such as 4-phenyl butyric acid (Longo et al., 2016). ER stress pharmacological inhibition can reverse metabolic dysfunction also in other tissues, including liver and brain (Ozcan et al., 2006; Longo et al., 2016).

Meta-inflammation and ER dysfunction are emerging as critical mechanisms. If these mechanisms are targeted therapeutically, they can enhance multiple metabolic parameters, as shown in preclinical and clinical studies (Hummasti and Hotamisligil, 2010).

### Dynamics of the ECM and Mechanical Stress

The protein composition and dynamics of the ECM are crucial for the adipocyte function. ECM remodeling is essential for the expansion and contraction of adipocytes to accommodate changes in energy stores (Rutkowski et al., 2015). During a positive energy balance, ECM accumulation occurs in AT, which contributes to fibrosis and impairs its role as a nutrient storage organ (Lee et al., 2014).

Abnormal accumulation of ECM components in AT has been shown to cause obesity-associated IR (Lin et al., 2016). Excessive ECM deposition in AT is suggested for triggering adipocyte necrosis, which attracts pro-inflammatory macrophages and causes AT inflammation and metabolic dysfunction. In addition, excess ECM deposition causes adipocyte death and AT inflammation by activation of integrins and CD44 signaling pathways (Lin et al., 2016).

Lipid accumulation occurring in obesity may also cause ECM instability and induce various mechanical stresses on these cells. The mechanisms governed by these mechanical stresses in adipocytes have not yet been fully explained, but certain pathways such as RhoA, and NF-κB have been evaluated. RhoA signaling, for instance, inhibits adipogenesis through *PPAR*γ suppression and stimulates the secretion of pro-inflammatory cytokines (McBeath et al., 2004; Li et al., 2010). Meanwhile, Li et al. (2010) have shown that the elevated density of ECM proteins reduces insulin signaling and increases MCP-1 secretion by activating the NF-κB signaling pathway (Li et al., 2010).

As mentioned above, some of the potential mechanisms involved in AT inflammation have been identified; however, it is likely that there are still unknown triggers. The temporal sequence of events leading to AT inflammation, as well as the contribution of each mechanism described above, has not yet been fully established. In our opinion, adipocyte hypertrophy may be the primary and initial event causing AT inflammation. In obesity, adipocytes respond to excess energy by storing lipids inside and undergoing dramatic changes in size (hypertrophy). Hypertrophy is associated with hypoxia, cellular and tissue stress, and cell death due to the activation of both necrotic and apoptotic mechanisms. Hypertrophic adipocytes are also characterized by excessive lipolysis, resulting in increased release of FFAs acting on TLR4, as previously indicated. All the above mechanisms promote adipocyte dysfunction, characterized by an altered cytokine secretion pattern. These mechanisms play a dual role; they are able both to trigger individually inflammatory responses and to induce downstream processes, amplifying and eliciting chronic systemic inflammation and thus promoting systemic IR. The temporal sequence of events suggested here and the relevance that we attribute to adipocyte hypertrophy in the initiation of AT inflammation needs to be further verified.

## Inflammation as a Therapeutic Target for Metabolic Diseases

The role of chronic inflammation, particularly in the AT, in the pathogenesis of T2D and associated complications, is now well established. The association between obesity, AT inflammation, and metabolic disease makes inflammatory pathways an appealing target to treat metabolic disorders. Inflammation is recognized as the pathologic mediator of these frequently common comorbidities. Several anti-inflammatory approaches have been tested in clinical studies of obese individuals with IR, but clinical trials to confirm the therapeutic potential are still ongoing (Goldfine and Shoelson, 2017). The number of available drugs that can target different components of the immune system and improve different metabolic aspects is increasing rapidly (Donath, 2014).

Based on the mechanism of action, therapeutic approaches to target inflammation in IR and T2D can be divided into (i) pharmacologic approaches that directly target inflammation and (ii) diabetes drugs with anti-inflammatory properties.

### Pharmacologic Approaches That Directly Target Inflammation: Salsalate

Salsalate is an analog of salicylate that belongs to the non-steroidal anti-inflammatory drug classes. Independent studies have shown that salsalate can improve glycemic control in T2D patients. The mechanism of action of salsalate in reverse hyperglycemia in obese mice is through the inhibition of NF-κB pathway and has been identified in 2001 by Shoelson (Yuan et al., 2001).

Goldfine then translated this initial finding to the clinical study and showed that salsalate decreases fasting glucose and triglyceride levels, increases adiponectin levels and glucose utilization in diabetic patients during hyperinsulinemic–euglycemic clamp, and improves insulin clearance (Goldfine et al., 2008). These observations have been confirmed in two multicenter, randomized, placebo-controlled trials in subjects with T2D (Goldfine et al., 2010, 2013). In the first study, treatment with this drug improves insulin sensitivity and decreases HbA1c levels by 0.5% relative to placebo over 14 weeks in a group of patients with T2D (Goldfine et al., 2010). In the second study, 48 weeks of salsalate administration in a larger patient population (283 participants; placebo, *n* = 137; salsalate, *n* = 146) leads to a smaller decrease in the levels of HbA1c (–0.33%) and serum triglycerides (Goldfine et al., 2013). This treatment also decreases levels of glycation end products (Barzilay et al., 2014).

Other studies also suggest that metabolic improvement, induced by salsalate treatment, is mediated through AMPK activation (Hawley et al., 2012). Although the effects on glycemic control are modest, the salsalate is not expensive and has a very safety profile.

### Pharmacologic Approaches That Directly Target Inflammation: TNF-α Inhibitors

In 1993, a preclinical study clearly showed the role of TNF-α in the pathophysiology of IR in the AT (Hotamisligil et al., 1993), and this finding has raised the hypothesis that TNF-α blockade has potential therapeutic benefits. However, the results of clinical studies have so far been disappointing. For instance, TNF-α neutralizing antibodies have been shown to be effective for the treatment of many other inflammatory diseases, and some patients have shown slight improvements in glycemic control (Ofei et al., 1996; Feldmann, 2002; Reilly et al., 2013). However, prospective studies in T2D patients have been confusing. In spite of valuable effects in mice, a human clinical trial showed that anti-TNF-α therapy leads to no improvements in insulin sensitivity in patients with T2D (Ofei et al., 1996; Moller, 2000; Paquot et al., 2000). In contrast, a study performed in obese subjects without T2D showed that an inhibition of TNF-α for 6 months is able to reduce fasting glucose and increase adiponectin levels (Stanley et al., 2011).

### Pharmacologic Approaches That Directly Target Inflammation: IL-1β Antagonists

IL-1β is a strong mediator of the obesity-induced inflammation and participates in the pathogenesis of T2D, mediating the adverse consequences of hyperglycemia on pancreatic β-cells (Maedler et al., 2002). Antagonism of IL-1R for 13 weeks, in a proof-of-concept study of patients with T2D, shows an improved glycemic control and secretory function of the pancreatic β-cells and the reduced markers of systemic inflammation (Larsen et al., 2007). The follow-up study on the same population proves that 39 weeks after the last IL-1R antagonist administration, β-cell insulin secretion is still increased and CRP decreased (Larsen et al., 2009). The long-term effects are probably due to the block of IL-1β auto induction mechanism (Böni-Schnetzler et al., 2008). Further studies have also noted that the use of antibodies directed against IL-1β has potential benefits in the treatment of T2D, as it significantly reduces HbA1c levels (Cavelti-Weder et al., 2012; Sloan-Lancaster et al., 2013).

Pathological activation of IL-1β also contributes to the development of other T2D-associated diseases, such as Crohn’s disease, gout, and RA (Donath et al., 2019). Recently, a multicenter randomized controlled trial, specifically designed to evaluate the glycemic outcome, enrolled participants, with RA and T2D (followed up for 6 months). Thirty-nine participants were randomized to IL-1R antagonist (anakinra) or TNF inhibitors (TNFi) to assess the efficacy of these drugs in controlling glucose alterations of T2D (Ruscitti et al., 2019). After 3 and 6 months of treatment, anakinra showed a significant improvement in metabolic alteration (reduction of HbA1c by more than 1%), whereas TNFi showed no enhancement. Regarding RA, there has been a gradual reduction in disease activity in both groups. In conclusion, results of this research indicate a specific effect of IL-1 inhibition in subjects with RA and T2D, reaching the therapeutic targets of both disorders and improving the main outcome of enrolled participants. A clearer reduction of HbA1c, comparing this to the previous study on T2D (Larsen et al., 2007), can be explained based on the theory that pathogenic mechanisms of T2D could be exaggerated in the context of RA. On this basis, IL-1 pathway can be considered a shared pathogenic mechanism, and a single treatment that manages both diseases appears to be a promising option for improving the care of RA and T2D patients (Giacomelli et al., 2016).

### Diabetes Drugs With Anti-inflammatory Properties: Thiazolidinediones

Thiazolidinediones (TZDs) are antidiabetic drugs that improve insulin sensitivity and glycemia, as they function as agonists for PPARγ nuclear receptor (Yki-Järvinen, 2004). TZDs have also anti-inflammatory effects; they repress NF-κB action and reduce the expression of its target genes (Pascual et al., 2005).

The inhibition of NF-κB pathway reduces ATM content (Esterson et al., 2013; Koppaka et al., 2013), restores the M2 macrophages phenotype (Chawla, 2010), and stimulates the recruitment of the anti-inflammatory regulatory T cells in the AT (Cipolletta et al., 2012).

Furthermore, the ability of TZDs to reduce circulating inflammatory mediators (such as CRP and MCP-1) seems to be independent of glycemic control (Pfützner et al., 2005). Therefore, TZDs act through different mechanisms and the anti-inflammatory properties of these drugs are not definitely established.

### Diabetes Drugs With Anti-inflammatory Properties: Metformin

The mechanism of metformin action is not completely explained, but it decreases glycemia by reducing hepatic glucose production and raising glucose uptake in peripheral tissues (Inzucchi et al., 1998). In addition to its clear metabolic effects, metformin has also anti-inflammatory properties; for instance, it directly inhibits the production of reactive oxygen species in the mitochondria and can reduce the production of many cytokines (Wheaton et al., 2014). Emerging evidence supports the novel hypothesis that metformin can exhibit immune-modulatory features. The effects of metformin on immune cells (T cells, B cells, monocytes/macrophages, neutrophils) involved in the pathogenesis of autoimmune and inflammatory diseases have been extensively reviewed by Ursini et al. (2018). Inside the immune cells, metformin temporarily inhibits the complex I of the mitochondrial electron transport chain, contributing to an increased AMP/ATP ratio (Rena et al., 2017). Decreased ATP concentration causes AMPK activation, and among several targets, AMPK inhibits the mammalian target of rapamycin (mTOR) (Zhou et al., 2001). mTOR is crucial for cellular metabolism, cytokine responses, antigen presentation, macrophage polarization, and cell migration (Weichhart et al., 2015) through its interaction with the STAT3 pathway (Saleiro and Platanias, 2015). Metformin can also regulate other pathways relevant to immune cells, including NF-kB (Hattori et al., 2006; Chaudhary et al., 2012) and JNK (Wu et al., 2011). Indeed, other studies have proved that metformin is able to inhibit TNF-α-induced activation of the NF-κB axis and IL-6 production (Huang et al., 2009) through PI3K-dependent AMPK phosphorylation. Metformin, in a dose-dependent manner, reduces IL-1β production in lipopolysaccharide-activated macrophages, and the effect is independent of AMPK activation (Kelly et al., 2015). Moreover, metformin concurrently decreases circulating inflammatory proteins, including CRP, in impaired glucose tolerance and T2D patients (De Jager et al., 2005; Haffner et al., 2005). The anti-inflammatory effects of metformin, like TZDs, appear to be independent of glycemic control (Caballero et al., 2004). In murine models, the attenuation of the inflammatory state has been shown to be effective in improving the obesity-induced IR; however, there are ongoing clinical trials in humans to confirm the therapeutic potential of metformin. This issue represents an essential step in proving the translational relevance of these observations.

T2D is a heterogeneous disorder, and the absence of clinical biomarkers, showing whether the treatments have anti-inflammatory effects in the AT, is a potential issue complicating the analysis (Donath, 2016). The identification and profiling of these biomarkers in T2D patients would allow us to predict those that should respond to an anti-inflammatory therapy.

## Conclusion

The global obesity epidemic results in a higher incidence of metabolic disorders. The mechanisms underlying the association between obesity and IR have not yet been fully explained. Therefore, further well-designed clinical and basic research studies are needed to establish this relationship. From our point of view, inflammation occurring in the AT during obesity is the primary mechanism for developing local and systemic IR. AT is the primary whole-body regulator of lipid and glucose homeostasis and is no longer considered merely a storage tissue.

Obesity leads to severe adipocyte disorders by altering the amount and activity of almost all resident immune cells. The imbalance of immunological phenotypes is correlated with the development of persistent local inflammation during which several biologically active molecules are released. These molecules affect distal tissues and organs, such as skeletal muscle and liver.

The inflammatory nature of obesity opens new prospects in the development of therapeutic strategies for the treatment of its related metabolic complications. However, there are still a lot of issues that need to be addressed.

Anti-inflammatory strategies have proven to be effective in improving obesity-induced IR in murine models. However, clinical studies are still ongoing to confirm the therapeutic potential in obese and insulin-resistant individuals. Another issue is the modest effects of anti-inflammatory therapies observed in these studies. Targeting only one inflammatory molecule may not be sufficient to have a beneficial effect; therefore, we could hypothesize the combined use of more anti-inflammatory therapies. In addition, a recent study showed that acute and transient inflammation is essential for healthy AT expansion and remodeling in obesity (Asterholm et al., 2014). This finding raises further questions on the effectiveness of anti-inflammatory therapies in the treatment of obesity-induced metabolic disorders. Inflammation is a finely regulated mechanism, and all defects in its balance can cause AT dysfunction.

In the era of personalized and precision medicine, increasing our knowledge of the obesity-induced inflammation mechanisms might enable us to overcome the limitations of the traditional anthropometric indices of obesity. These anthropometric indices are not correlated with obesity-induced metabolic complications and additional clinical parameters need to be identified for risk assessment (Longo et al., 2019). From our point of view, given the strong association between inflammation and obesity complications, circulating inflammatory biomarkers may be used for the risk assessment of these diseases in the future. The identification and evaluation of these biomarkers in obese patients will allow the prediction of those who will develop obesity-associated metabolic complications.

## Author Contributions

FB and CM conceived the idea and edited the manuscript. FZ, ML, JN, GR, and AD wrote the manuscript. FZ and ML prepared the figures. All authors reviewed the manuscript.

## Conflict of Interest

The authors declare that the research was conducted in the absence of any commercial or financial relationships that could be construed as a potential conflict of interest.

## Abbreviations

AMPK: AMP-activated kinase
AT: adipose tissue
ATMs: adipose tissue macrophages
CRP: C-reactive protein
DAMPs: danger-associated molecular patterns
DCs: dendritic cells
ECM: extracellular matrix
ER: endoplasmic reticulum
Flt3L: Fms-like tyrosine kinase 3 ligand
HbA1c: hemoglobin A1c
HFD: high-fat diet
HIF-1: Hypoxia-inducible factor-1
HREs: hypoxia-response elements
IFN-γ: Interferon-γ
IKKβ: IκB kinase β
IL-18: Interleukin-18
IL-1R: IL-1 receptor
IL-1β: Interleukin-1β
IL-6: Interleukin-6
ILC2s: innate lymphoid cells
iNKTs: Invariant natural killer T cells
IR: insulin resistance
IRS-1: insulin receptor substrate-1
IRSs: insulin receptor substrates
IκBs: inhibitors of κB
JNK: C-JunN-terminal kinase
MAPK: mitogen-activated protein kinase
MCP-1: Monocyte chemotactic protein-1
NF-κB: nuclear factor kappa-light-chain-enhancer of activated B cells
NKs: natural killer cells
NKTs: Natural killer T cells
NLRs: nod-like receptors
PAMPs: pathogen-associated molecular patterns
PI3K: phosphatidylinositol 3-kinase
PKB: protein kinase B
PPARγ: Peroxisome proliferator-activated receptor γ
RA: Rheumatoid arthritis
RhoA: Ras homolog gene family member A
T2D: Type 2 diabetes
TGF-β: Transforming growth factor-β
Th1: T helper 1 cells
TLRs: Toll-like receptors
TNF-α: Tumor necrosis factor-α
TNFi: Tumor necrosis factor inhibitors
Tregs T: regulatory cells
TZDs: Thiazolidinediones
UPR: unfolded protein response
WAT: white adipose tissue.
